# A molecular survey and phylogenetic analysis of porcine circovirus type 3 using oral fluid, faeces and serum

**DOI:** 10.1186/s12917-020-02489-y

**Published:** 2020-08-10

**Authors:** Jan Plut, Urska Jamnikar-Ciglenecki, Irena Golinar-Oven, Tanja Knific, Marina Stukelj

**Affiliations:** 1 Clinic for Ruminants and Pigs, Clinic for Reproduction and Farm Animals, Veterinary Faculty, University of Ljubljana, Ljubljana, Slovenia; 2 Department of Food Safety, Institute of Safe Food, Animal Nutrition and Environment, Veterinary Faculty, University of Ljubljana, Ljubljana, Slovenia; 3 Institute of Microbiology and Parasitology, Veterinary Faculty, University of Ljubljana, Ljubljana, Slovenia

**Keywords:** Viral disease, Pig oral fluid, Faeces, Serum, PCV3, PCR, First report, Slovenia

## Abstract

**Background:**

Porcine circovirus type 3 is the most recently discovered porcine circovirus, and an emerging pathogen. In this study the status of its presence on some Slovenian farms is reported. The effectiveness of the vaccine against porcine circovirus type 2 was assessed against porcine circovirus type 3.

Group samples of oral fluid, faeces and individual serum samples were taken from six different pig categories and tested for presence of viral DNA, using both real time and conventional PCR. Positive samples were subjected to direct Sanger sequencing. Nucleotide sequences were analyzed and compared to GenBank PCV3 sequences.

**Results:**

Positive samples were sent for genome sequencing, which confirmed the presence of virus in all different pig categories on five farms. A high to moderate correlation of strong statistical significance was found between individual serum samples, oral fluid and faeces. Slovenian PCV3 was found to be distributed in a way similar to that of other countries. Slovenian PCV3 nt sequences are highly related, sharing more than 99.5% nt identity. On one farm a commercially available vaccine against porcine circovirus type 2 was used on 3-week-old pigs. It did not affect the presence of porcine circovirus type 3 in oral fluid or sera of any of the seven age groups of pigs, each with two control groups.

**Conclusions:**

The results constitute the first discovery of the virus in Slovenia. Genome sequencing has revealed a high degree of similarity between Slovenian and GenBank isolates.

## Background

Porcine circovirus type 3 (PCV3) is the most recent of this virus types to be discovered, first in the USA [1] then globally, including South America [2], Asia [3, 4] and Europe, namely Poland [5], Germany [6], Italy [7] and Spain [8]. Together with non-pathogenic type 1 (PCV1) [9] and potentially pathogenic type 2 (PCV2), which is one of the leading causes of severe economic loss in swine production [10], PCV3 is a member of the *Circoviridae* family and the *Circovirus* genus [11]. It is considered to be an emerging disease causative agent, although its pathogenic effect is yet to be resolved [8]. Retrospective studies show that the virus has been present since the early 90s [12, 13].

PCV3 has a non-enveloped virion with an icosahedral structure containing a single-stranded DNA [14] and a genome of 2000 bp, the largest of the three PCVs. The genome contains two major open reading frames, ORF1 and ORF2, encoding replicase (Rep) and capsid (Cap) proteins respectively [2]. A further open reading frame for PCV3 has been suggested but its start codon has still not been determined [6]. The 5′ end of PCV3 contains 235 nucleotides (nt) and is identical to regions previously discovered in PCV1 and PCV2 [15]. Most recent phylogenetic analyses suggest that its sequences can be divided into two groups, PCV3a and PCV3b with, respectively, more than 99.1 and 97.3% nucleotide sequence identities, which can be subdivided into subgroups, including: a1, a2, b1 and b2 [6]; PCV3a, PCV3b and PCV3-IM [16]; PCV3a, PCV3b and PCV3c [17]; and PCV3a-1, PCV3a-IM, PCV3a-2 and PCV3b [18].

Several clinical conditions have been linked to PCV3, most of them resembling those caused by PCV2. These are porcine dermatitis and nephropathy syndrome (PDNS), post-weaning multisystemic wasting syndrome (PMWS), reproductive failure, cardiac and multisystemic inflammation [1], respiratory disorders [19] and congenital tremor [20]. PCV3 is found in pigs of all ages. Most of the cycle threshold (Ct) values obtained by quantitative PCR are higher than 30 (Ct > 30) [5–7, 19] and raise doubts about PCV3’s pathogenic potential. On the other hand, this may suggest that infection is mostly subclinical or only clinically visible when co-factors enhance the primary effect of PCV3 [12]. The genomes of PCV2 and PCV3 are identical up to 37% and this raises questions concerning possible cross-immunogenicity [21]; in that case, anti-PCV2 vaccination should also eliminate or reduce PCV3 virus burden [15, 22]. Since swine circovirus associated diseases are a problem faced by Slovenia’s pig producers [23], we considered it likely that PCV3 was also present on Slovenian farms.

The vaccine preventive program on each farm in Slovenia depends on the state of the health of each estate. Anti-PCV2 vaccines are one of the commercial vaccines most often used, due to the wide presence of circovirus-associated diseases in swine herds and to the strong efficacy of these products which improve the health status of the herd and the economic situation. Slovenian farmers most often vaccinate 21-day-old suckling piglets against PCV2 to eliminate clinical signs during weaning. This could affect the presence of PCV3.

According to the Republic of Slovenia Statistic Office, Slovenian gross production of domestic pigs is one of the smallest in Europe. There are currently 230.884 pigs, 25.494 of which are breeding sows. There are 12.263 small-sized pig farms in Slovenia that own from 1 to 20 pigs each. On the other hand, there are only 34 farms with 500 to 1.000 pigs and 16 farms with more than 1.000. All 6 farms in this study are from the last two size groups, including two of the largest Slovenian farms with 3.000 breeding sows. These pigs derive mostly from national nucleus breeding facilities. Most of the Slovenian farms do not import live animals from abroad and never directly from countries that are not part of the European Union. Of those imported, Slovenian breeders mostly purchase animals from northerly neighbouring country Austria or from Germany. The largest farm uses boars and gilts bought in Denmark. On Farms 1 and 2, all newly purchased animals go through 30-day quarantine on remote locations. Farms 4 and 5 apply 60-day quarantine period on remote locations for all newly purchased pigs, and perform diagnostic at the beginning and the end of the period. Farm 4 weekly supplies Farm 5 with replacement gilts that go through a 90-day acclimatization period for PRRSV and other endemic diseases. Farms 3 and 6 do not have a quarantine period for their newly purchased pigs.

The main objectives of this study were to establish the presence of PCV3 on Slovenian farms and to compare the sequences of their genome to GenBank PCV3 sequences. Furthermore, we aimed to assess the efficiency of isolation of the viral DNA from oral fluid (OF) samples, faeces samples, and from individual serum samples and the efficiency of the molecular detection methods for identifying PCV3 in them, as well as to determine whether anti-PCV2 vaccination protects against PCV3.

## Results

### Real-time PCR and PCR on OF and faeces group samples and on individual serum samples

Results are divided according to the methodology applied; Ct values for real-time PCR, the result of conventional PCR testing of group OF and faeces samples and individual sera samples are listed in Table 1; a numerical value represents a cycle-threshold where a real-time PCR signal was detected. Plus or minus symbol in the cell indicates a positive/negative result with PCR. The Farm 3 is left out from the Table 1, since all results of the tested samples were negative. Most of the results with Ct levels less than 37 cycles were also positive with conventional PCR (marked as plus symbol in Table 1). When Ct levels were over 37, conventional PCR mostly gave negative results ( minus symbol in Table 1). There were, however, two cases where values of Ct were less than 37 and conventional PCR was negative and vice-versa; all these being detected on Farm 6.

**Table 1:** Real-time PCR and conventional PCR results in group samples on farms where PCV3 was detected

			***pig category***														***Total samples by farm (pos./tested)***^***a***^	
			5w		7w		9w		11w		13w		fatteners		b. sows		*OF*	*sera (pos./all)*
																	*faeces*	
***farm no.***	**1**		*34.95* *+*	*2/10*	*34.5* *+*	*3/10*	*37.44* *-*	*0/10*	*36.54* *+*	*0/10*	*38.19* *-*	*0/10*	*37.84* *-*	*0/10*	*33.68* *+*	*5/10*	*7/7*	*10/70*
			*neg.*		*neg.*		*neg.*		*neg.*		*neg.*		*neg.*		*neg.*		*0/7*	
	**2**		*neg.*	*0/10*	*neg.*	*0/10*	*neg.*	*0/10*	*neg.*	*0/10*	*neg.*	*0/10*	*39.31* *-*	*0/10*	*neg.*	*0/10*	*1/7*	*0/70*
			*neg.*		*neg.*		*neg.*		*neg.*		*neg.*		*neg.*		*neg.*		*0/7*	
	**4**		*30.39* *+*	*4/10*	*32.52* *+*	*4/10*	*38.34* *-*	*0/10*	*neg.*	*0/10*	*32.07* *+*	*2/10*	*neg.*	*0/10*	*31.85* *+*	*3/10*	*5/7*	*13/70*
			*37.00* *-*		*36.1* *+*		*neg.*		*neg.*		*36.38* *-*		*neg.*		*37.04* *-*		*4/7*	
	**5**		*37.72* *-*	*0/10*	*33.54* *+*	*0/10*	*39.16* *-*	*0/10*	*38.23* *-*	*0/10*	*neg.*	*0/10*	*38.11* *-*	*0/10*	*39.7* *-*	*0/10*	*6/7*	*0/70*
			*neg.*		*38.37* *-*		*neg.*		*neg.*		*neg.*		*neg.*		*neg.*		*1/7*	
	**6**		*27.96* *+*	*3/8*	*N/A*	*N/A*	*33.42* *+*	*3/9*	*37.95* *+*	*2/10*	*36.41* *-*	*0/10*	*35.32* *+*	*0/10*	*37.49* *+*	*0/10*	*6/6*	*8/57*
			*32.9* *+*		*N/A*		*36.45* *-*		*neg.*		*neg.*		*neg.*		*38.2* *-*		*3/6*	
***Total samples/category***	*OF*	*Sera (pos./all)*	*4/5*	*9/48*	*3/4*	*7/40*	*4/5*	*3/49*	*3/5*	*2/50*	*3/5*	*2/50*	*4/5*	*0/50*	*4/5*	*8/50*	***25/34 (73.5%)***	***31/237 (13.1%)***
	*faeces*		*2/5*		*2/4*		*1/5*		*0/5*		*1/5*		*0/5*		*2/5*		***8/34 (23.5%)***	

The numerical value in OF/faeces cell represents a Ct value obtained with real-time PCR.

The symbol in the OF/faeces cell represents the result of conventional PCR testing (**+** = positive, **−** = negative, [no symbol] = not tested),

The right part of the cell represents a number of positive individual sera compared to all tested sera with conventional PCR testing.

N/A = category not available for testing at the farm.

*neg* negative.

*pos* positive.

^a^all the samples from Farm 3 are PCV3 negative farm are excluded from the table

Overall, real-time PCR signalled positive in 73.5% of all 34 OF samples tested, in 23.5% of faecal samples, and in 13.1% of all 237 tested individual sera samples. Detectable Ct values were generally obtained in all animal categories, this being confirmed with conventional PCR. However, among pigs of all ages, there was only one sample where virus was confirmed in fatteners, using conventional PCR, i.e. in OF on Farm number 6. Viral DNA was not detected in any of their individual sera. In general, the viral DNA was found in almost all younger weaners (5 and 7 w/o) in all three tested samples. As animals get older, the number of positive cases started to drop slightly, especially the number of viremic pigs. In fact, all sera of fatteners were negative. At the farm level, virus could be detected in all age categories on positive farms, or in none, as in the case of Farm number 3. Only on farm number 2, did real-time PCR give a positive result, and then only in OF of fatteners. This, however, was not confirmed with conventional PCR; no other samples from that farm were positive. In farm number 2, anti-PCV2 vaccination with a commercial vaccine was used, as was the case for the PCV3 positive Farms 1 and 5. Viral DNA was not always detected by conventional PCR, when Ct values were obtained using qPCR. Fisher’s exact test proves association between real-time PCR results for faeces and OF and PCR results for individual sera. Agreement between real-time PCR for faeces and OF is moderate (K = 0.48), but acceptable or good regarding individual sera and faeces, K = 0.57 and K = 0.67 respectively. Faeces and individual sera were moderately correlated (Spearman’s rho = 0.51) and strongly statistically significant (*p*-value = 0.0019).

### Detection of PCV3 DNA in weaners vaccinated with commercially available anti-PCV2 vaccine

Despite anti-PCV2 vaccination, PCV3 DNA was detected in OF from seven of the nine tested groups of pig animals (7.8%). One of the two groups of unvaccinated 5 week-old (w/o) tested positive for the presence of PCV3 DNA in OF, with three out of four and three out of three groups of 6 and 8 w/o weaners’ OF testing positive for PCV3 DNA respectively. Results of the vaccination are presented in Table 2. Vaccinated or not, PCV3 was absent from the serum of all but one group of 6 w/o weaners (11.1%). No visible clinical signs associated with PMWS or PDNS were observed in any of the animals.

**Table 2:** Detection of PCV2 and PCV3 in weaners aged 5, 6 or 8 weeks after intramuscular application of commercial anti-PCV2 vaccine at week 3 on farm *no.5*

	PCV2		PCV3	
	OF	serum	OF	serum
5 w/o unvaccinated	*pos.*	*neg.*	*neg.*	*neg.*
5 w/o unvaccinated	*neg.*	*neg.*	*pos.*	*neg.*
6 w/o vaccinated	*neg.*	*neg.*	*pos.*	*pos.*
6 w/o vaccinated	*pos.*	*neg.*	*pos.*	*neg.*
6 w/o vaccinated	*pos.*	*neg.*	*pos.*	*neg.*
6 w/o vaccinated	*neg.*	*neg.*	*neg.*	*neg.*
8 w/o vaccinated	*pos.*	*neg.*	*pos.*	*neg.*
8 w/o vaccinated	*neg.*	*neg.*	*pos.*	*neg.*
8 w/o vaccinated	*pos.*	*neg.*	*pos.*	*neg.*

The detected DNA with conventional PCR in OF or sera samples in 9 different pig groups depending on the vaccination

### Phylogenetic analysis

**Fig. 1:**
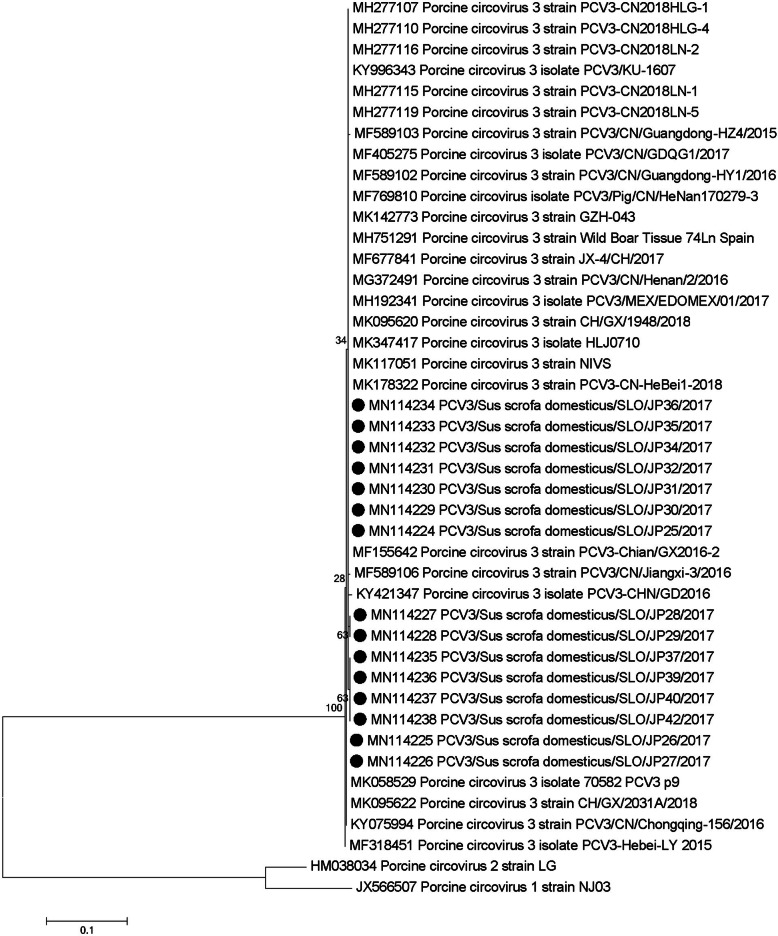
Phylogenetic analysis and relationships between PCV3 isolates. The phylogenetic tree is based on the isolation and sequencing of a 455 bp nucleotide DNA fragment located in the PCV3-*rep* region. Highly related isolates (more than 99.5% nt identity) are shown

Phylogenetic analysis of a replication-associated protein gene was performed and nt sequence identities calculated to determine Slovenian PCV3 strain genetic relationships. Slovenian and other GenBank PCV3s were then analyzed and compared. Slovenian PCV3 was found to be distributed in a way similar to that of other countries on the ML phylogenetic tree; Slovenian PCV3 nt sequences are highly related, sharing more than 99.5% nt identity; and Slovenian PCV3 sequences are highly related or identical to GenBank PCV3 sequences (Fig. 1).

## Discussion

PCV3 has been rapidly and globally detected since first being discovered [1], raising the question of whether it should be considered as an emerging pathogen. Much has been speculated about its pathogenicity [1, 8, 19, 20], but our review of these studies indicates that no clear connection between viral presence and clinical signs has been established indisputably. It is possible that a high viral load in tissue is associated with PCV2-like multisystemic failure, respiratory disease and PDNS [1, 19]. A comprehensive study of PCV3’s molecular pathogenicity and the immune response to it is necessary before a definite final statement can be made.

Our discovery of PCV3 in Slovenia, the first of its kind, came as no surprise given the global situation. We collected and studied three different sample types for isolating and detecting PCV3 DNA, each with a different degree of usefulness for molecular detection of the virus. Viral DNA was detected using both qPCR and conventional PCR, and a correlation was established. The threshold obtained and presented as a qPCR Ct value was approximately 37 cycles, in accord with that presented in previous studies [5, 7]. There were exceptions: conventional PCR confirmed virus presence in OF samples, but disproved it in some of the faeces samples; this finding is in accordance with previously noted studies - samples with Ct values around 37 appear to be in the grey area between positive and negative. Farm 2 stood out among the others; all except the OF sample of fatteners tested negative. Conventional PCR did not support the real-time PCR result, so Farm 2 might even be considered as PCV3 negative. PCV3 DNA from the same animal group was more often found in OF than in faeces, and Ct values from faeces were generally higher, suggesting that less virus is shed by faeces than by OF, as seen in Table 2. OF is thus a better medium for detecting PCV3 DNA than faeces, with the percentages of positive samples significantly higher, comparing 73.5% positive OF samples to 23.5 and 11.3% positive faeces and sera samples respectively. Judging from the number of positive samples from each pig category, weaners acquire and carry the viral DNA, whereas animals become free of the virus when getting older; the numbers of PCV3 positive fatteners are the lowest. Surprisingly, virus can later again be found in breeding animals in numbers comparable to those in younger weaners. In the future, it would be interesting to assess the immunity status of the different pig categories, in correlation with the presence of virus.

Using Fisher’s exact test for statistical analysis, we established a moderate to strong correlation between all the samples used for the detection of viral DNA; the correlation and statistical significance was strong and very significant for faeces and individual sera respectively but, taking into account that viral DNA was more often found in OF than in other samples, that would suggest OF to be the sample in which PCV3 can be detected at most times. The lower number of positive samples detected in faeces than in OF can also be a consequence of the large number of inhibitory substances present in faeces [24, 25]. However, that was already taken into account during the optimisation of the protocol prior to detection in this study.

According to the results during the optimisation process, validation of the method was also performed. Conventional PCR was used to detect PCV3 DNA in individual serum samples taken from the same group of animals. Samples were taken from, and the virus only found in, animals whose real-time PCR group sample Ct values were lower than 37.00. However, viral DNA was detected in 50% of one group of individual samples, indicating that not all of these animals developed viremic states. This means that a general rule cannot be firmly established. This also raises questions about the individual factors responsible for this situation and about the usefulness of individual serum sampling for screening, since its prevalence was moderately low (< 50%) in some group samples. We detected the virus in all animal categories, as had been the case for several other authors [5, 20], but no clear correlation was found between age and animal category prevalence.

The same primers and probes for viral DNA extraction and detection were used as those developed and validated for both conventional PCR and qPCR methodology [7]. Viral DNA tested in our laboratory only covers the ~ 500 bp product in the PCV3-*rep* region of the genome so cannot be fully compared with that studied by the authors who sequenced the complete genome. Fifteen genomes were isolated and sequenced in Slovenia, showing a 99.5% similarity inside its borders. Our virus strain is ~ 99.5–100% complementary to China’s MK347417 PCV3 HLJ0710 [16], MF583106 PCV3/CN/Jiangxi-3/2016 [18] and KY075994 PCV3/CN/Chongqing-156/2016 [26]. On the other hand, Faccini et al. in 2017 only showed 99.2% similarity [27] when compared to the Chinese strain discovered by Ha et al. [26]. The close similarity between Slovenian and Chinese strains could come as a surprise since Slovenian farms do not import animals from China; but the close relationship to clusters from the far East has been observed throughout Europe. General variability inside the genome of PCV3 seems to be very low where, for example, the Brazilian strain is the least related to the KY418606_China strain, but that similarity is still at ~ 97% [2]. Compared to this, genome variability inside the genome of the longer known PCV2 is bigger. Similarly, very low intragenome diversity and high similarity to far East strains was confirmed in the genome studies of German PCV3 strains by Fux et al. in 2018 [6]. In our opinion this shows the genetic stability of the virus population, which might point to the assumption that the PCV type 3 is in fact a more ancient, though only recently discovered, virus. Such a strong similarity can point towards the fact that the PCV3 has been present worldwide for a longer period of time though not detected as such. Due to the speculatively very similar clinical signs to the type 2 virus relative, infection by PCV3 might have been falsely correlated to PCV2 virus.

Subclusters have only been suggested by several authors who have compared PCV3’s complete genomes; if our partial genome isolates are compared to their closest relatives, our sequences could be placed in either the PCV3a-IM subcluster or the PCV3b subcluster. It had been suggested that PCV2-PCV3 cross-protection might occur [21], though we did not expect it, nor was it found to occur.

Woźniak et al. reported a slight reduction in PCV3 and circovirus associated clinical signs after vaccinating 3–4 w/o suckling piglets, but this was not supported statistically [21]. We also vaccinated and tested a small group of animals in which PCV3 was discovered on Farm 5 and found that, while the number of PCV2-positive samples fell slightly, no noticeable change in PCV3 presence occurred. The deductions from this thesis should be further explored using a larger sample than that used here.

## Conclusion

Our study confirmed the presence of PCV3 virus in Slovenian pig herds. The evidence showed a high degree of genotype similarity within Slovenia and to other PCV3 strains globally. Of the OF, faeces and sera samples in which the virus is detected, OF has proved to be the best for viral DNA isolation and detection. Our results suggest that the commercially available anti-PCV2 vaccines most probably do not act against PCV3.

## Methods

### Animals and farms

The samples were taken as a part of a survey in a national project from year 2016, and included the detection of some pathogens, but not PCV3, which was supposedly not present at the time. The samples were then examined retrospectively, since the PCV3 was discovered as a potentially important disease worldwide. Samples were taken at six different pig farms, two large one-site farms with 3000 breeding sows, a two-site farm with approximately 500 breeding sows, and three small one-site farms with less than 100 breeding sows. The pigs were grouped in the following age bands: 5 weeks-old (w/o); 7 w/o; 9 w/o; 11 w/o; 13 w/o weaners; fatteners; and breeding sows. The selected farms were considered as one of the biggest on our territory and generally well organized. Biosecurity status on these farms was considered medium to high level with sufficient documentation and information available on animal movement and production data. Smaller farms and backyard farms were excluded from the study as the number of in-housed animals was too small and not all pig categories were available on a single farm.

### Sampling

Prior to the sampling and testing, the status of PCV3 on all 6 farms in this study was unknown. Group samples of faeces and oral fluid and individual serum samples were used for comparison in order to determine which is the most useful for viral DNA detection. Age groups and sizes were organized in such a way that the results could be compared between farms. Most of the groups in each category consisted of 10 pigs, except for Farm 6, the smallest in size, in which some batches of weaners comprised fewer than 10 pigs, and 7 w/o weaners were absent. Number of pigs in each age group was not calculated, instead Farm 6, the smallest farm, was used as a common denominator. On other farms, where the number of pigs might exceed 10, sampling was randomized with R Statistical Software, version 3.6.0 (Foundation for Statistical Computing, Vienna, Austria) random number generator. Randomly selected 10 pigs were then sampled individually and a group OF sample was collected. Altogether, 40 group samples of oral fluid and faeces and 295 individual serum samples were collected (Table 3); 34 group oral fluid and faeces samples and 237 individual sera samples underwent testing, providing results for the purposes of our study.

**Table 3:** The number of samples collected at each farm from each pig category

		number of collected samples		
		***OF***	***faeces***	***serum***
farm number	***1***	7	7	70
	***2***	7	7	70
	***3***	6	6	58
	***4***	7	7	70
	***5***	7	7	70
	***6***	6	6	57
*total*:		**40**	**40**	**395**

### Sample preparation

Ten individual blood samples were drawn from the anterior vena cava from each group of animals. Group samples of oral fluid were obtained from the same groups from which blood was drawn. Cotton ropes provided in an IDEXX Oral Fluid Collection Kit were used. The ropes hung for half-an-hour above an open spot in the middle of pig pens, away from feed and drinking water. The collected oral fluids were stored in sterile 50 ml screw cap plastic containers. Group samples of fresh faeces were also collected from the same pigs from random pen sites and stored in sterile 100 ml screw cap plastic containers, together with a lesser amount taken directly from the recta. Samples were transported to the laboratory in a refrigerated box at 4 °C. Oral fluid samples were centrifuged for 10 min at 2000×*g* and the resulting supernatant stored at − 70 °C. A 10% suspension in RPMI-1640 (Thermo Fisher Scientific, Carlsbad, CA, USA) was prepared from group faeces samples; suspensions were centrifuged at 2000×*g* for 10 min before transferring the supernatant to sterile 20 ml screw cap plastic containers and storing at − 70 °C before further testing. Sera were centrifuged for 10 min at 3000×*g* after the formation of coagula. These samples were stored individually in 20 ml sterile cryotubes at − 70 °C.

### Testing scheme for each farm and for each category of animal

All oral fluid and faeces group samples from each category of pigs on all farms were first tested with real-time PCR and the Ct values were thus obtained. Wherever this methodology gave a positive result, the samples were re-tested with conventional PCR followed by gel electrophoresis. In the second part, from the positive tested animal categories determined by the initial testing, all the pigs from the group were tested individually. All group samples were tested with both real-time and conventional PCR.

### Extraction of nucleic acid

Nucleic acids were extracted manually, using the QIAamp Viral RNA Mini Kit (Qiagen, Germany), according to manufacturer’s instructions. DNA was extracted from 140 μL of the supernatant and eluted in 60 μL of elution buffer.

### PCV3 DNA detection

Real-time PCR (qPCR) was first used to detect PCV3 DNA in oral fluid and faeces group samples. Positive group samples were then retested using PCR; if PCR further confirmed the presence of PCV3 DNA, ten individual serum samples from the said groups, fewer if sufficient animals were not available, were tested using PCR.

Real-time PCR was performed with the FastStart Universal Master Quantitative PCR System (Roche). A 25 μL final reaction of 12.5 μL master mix, 7.25 μL DNase/RNase-free water, 1.125 μL of each primer (PCV3 353F and PCV3 465R at 20 pmol/μL concentration, containing sequences 5′-TGA CGG AGA CGT CGG GAA AT-3′ and 5′-CGG TTT ACC CAA CCC CAT CA-3′, respectively), 1 μL of qPCR-PCV3 probe (sequence: FAM-GGG CGG GGT TTG CGT GAT TT-BHQ1 in 5 pmol/μL concentration) [7] and 2 μL of the extracted DNA being analyzed. Reaction was performed with QuantStudio3 (Thermo Fisher Scientific, Massachusetts, USA) under thermocycling conditions of 2 min at 50 °C and 10 min at 95 °C, followed by 45 cycles of denaturation at 95 °C for 15 s, and annealing at 60 °C for 1 min as was originally described by Franzo et al. in 2018 [7].

Platinum PCR SuperMix (Invitrogen) was used to detect PCV3 DNA with a classic gel electrophoresis. A 25 μL reaction of 21.5 μL Platinum PCR SuperMix, 0.75 μL of each primer (PCV3 233F and PCV3 718R in 20 pmol/μL concentration, containing sequences 5′-AAA GCC CGA AAC ACA GGT GGT GT-3′ and 5′-TTT TCC CGA CAT CCT GGA GGA CCA AT-3′) [7] and 2 μL of DNA being analyzed, with a Mastercycler Nexus Gradient (Eppendorf, Germany). Thermal conditions were 2 min at 94 °C, followed by 35 cycles of denaturation at 94 °C for 30 s, annealing at 68 °C for 30 s, elongation at 72 °C for 1 min, followed by final elongation at 72 °C for 7 min. A 483 bp amplified PCR product was visualised in a 1.8% (w/v) agarose gel.

### PCV3 genome sequencing

The PCR product was electrophoresed in a 1.8% agarose gel and deemed positive according to the expected DNA fragment size. It was subjected to direct Sanger sequencing with prior purification by Macrogen (The Netherlands). The resulting 455 bp nucleotide sequences were analysed using DNASTAR’s Seqman and EditSeq (Lasergene, WI, USA), and compared with GenBank (NCBI) PCV3 sequences, using BLASTn; multiple alignments were created using *MEGA 7.0.21* [28]; and the nucleotide substitution model with the lowest BIC score was deemed the best fit. Phylogenetic trees were constructed using the ML method and Kimura 2-parameter model (K2). Statistical support for the phylogenetic tree was evaluated using 1000 repetition bootstrapping. Sequences were deposited in GenBank under accession numbers MN114224 to MN114238.

### Application of commercial anti-PCV2 vaccine on farm 5

After detecting PCV3 on Farm 5, the second largest Slovenian farm, a batch of three-week-old suckling piglets was vaccinated with a commercially available anti-PCV2 vaccine to assess its impact on PCV3. Four-week-old suckling piglets were weaned and divided into groups of 30. Samples taken for conventional PCR testing two, three and 5 weeks after vaccination were taken from three groups of 30 weaners when they were six and 8 weeks old. As controls, samples from two groups of non-vaccinated five-week-old weaners were used. Clinical status was examined for signs of circovirus related disease.

### Statistical analysis

Results from Real-time PCR on faeces, oral fluid and sera were tested for association by Fisher’s exact test, and methodological agreement between these methods was quantified with Kappa statistics. We also calculated Spearman’s rank correlation coefficients, choosing the non-parametric coefficient because the Shapiro-Wilk normality test showed that variables were not normally distributed. *P*-values less than 0.05 were considered statistically significant for all statistical testing. Statistical analysis was performed using R Statistical Software, version 3.6.0 (Foundation for Statistical Computing, Vienna, Austria).

## Data Availability

The datasets generated and/or analysed during the current study are available in the National Center for Biotechnology Information (NCBI) repository https://www.ncbi.nlm.nih.gov/popset?DbFrom=nuccore&Cmd=Link&LinkName=nuccore_popset&IdsFromResult=1798058592. The data about the participant farms are not publicly available due to privacy or ethical restrictions, other data in this study are available upon request from the corresponding author.

## Abbreviations

Ct: Cycle threshold
OF: Oral fluid
ORF: Open reading frame
PCR: Polymerase chain reaction
qPCR: Real-time polymerase chain reaction
PCV2: Porcine circovirus 2
PCV3: Porcine circovirus 3
PDNS: Porcine dermatitis and nephropathy syndrome
PMWS: Post-weaning multisystemic wasting syndrome
